# PW02-014 - Long term outcome and quality of life in TRAPS

**DOI:** 10.1186/1546-0096-11-S1-A154

**Published:** 2013-11-08

**Authors:** T Lane, A Bybee, NM Stewart, DM Rowczenio, JD Gillmore, R Al-Nackkash, H Trojer, AD Wechalekar, PN Hawkins, HJ Lachmann

**Affiliations:** 1 National Amyloidosis Centre, University College London, London, UK

## Introduction

TRAPS is a rare autosomal dominant disease. The long term outcome in adults has been relatively little studied.

## Objectives

We describe a cohort of 80 patients with sequence variants in *TNFRSF1A* who attended our centre for diagnosis and management.

## Methods

We examined medical and laboratory records for investigation and test results including genotype, phenotype, treatments and treatment responses, and organ function.

## Results

Between 2000 and 2012, 80 individuals with sequence variants and clinical disease were identified: 26 (33%) with one of the pathogenic variants of undetermined significance, R92Q or P46L; and 54 (67%) with one of a variety of other known or novel pathogenic variants. 32 (40%) were male, and 42 (53%) had a positive family history.

Of the 54 patients with a pathogenic sequence variant, 21 (39%) were not on long term treatment: 15 did not have persistent symptoms and so were treated with intermittent steroids (5 with intronic/splice junction mutations, 2 C33Y, and 1 each having H66L, D12E, C55Y, H22R, C43G, V83M, C88Y, C96Y); 1 patient (T50M) was refractory or intolerant of other treatments; 5 patients elected not to have treatment (1 each with C29F, C33Y, H22Q, D42del, T50M).

33 (61%) patients were on long term treatment for persistent and/or disabling disease symptoms: 29 (88%) on anti-IL-1 agents, 3 (9%) on anti-TNF and 1 (3%) on daily steroid therapy. 19 were treated with anakinra (4 C33Y, 3 T50M, 2 H22R and D42del, and 1 each of R92P, I57S, C55Y, H22Q, C30Y, T37I, F60L, and 1 with a novel in-frame deletion of 24 nucleotides [c.255_278del] in exon 3); all but 1 experienced a complete remission (CR) of disease. 10 patients were treated with canakinumab (2 C33Y, 2 T50M, and 1 each with C30R, Y38S, C43G, D42del, T37I, F60L); 9 have had a CR and 1 patient was not evaluable. 3 were treated with etanercept, 1 (C33Y) with CR and 2 (1 with T50M and the other with intronic substitution c.626-32G>T in intron 6) having intermittent steroids as well with only partial remission (PR, defined as good but incomplete resolution of symptoms or serum inflammatory markers). 1 patient (C33Y) with severe disease proving refractory to other therapies was treated with steroids 30mg daily, achieving PR.

Health-related quality of life (QoL) was surveyed in 29 patients with a pathogenic sequence variant, 10 who were treated only with intermittent steroids as their phenotype was not severe enough to warrant use of biologics, and 8 were surveyed before and after commencement of biologic therapy. In the latter group, post-treatment scores in physical, role and social functioning and bodily pain, general health and vitality were all greatly improved from prior to treatment. Interestingly, those who were only treated with intermittent steroids showed scores comparable with the post-treatment scores of the group treated with biologics, showing the impact of the untreated, more severe phenotype on QoL.

## Conclusion

This cohort shows the great variability in genotype and phenotype associated with sequence variants in *TNFRSF1A.* Those with a less severe phenotype may enjoy a good quality of life, and this may also be achieved by those with more severe disease provided they are being treated effectively.

## Disclosure of interest

None declared.

